# Procedural Trends in Catheter Ablation of Persistent Atrial Fibrillation: Insights From the Real-AF Registry

**DOI:** 10.1161/CIRCEP.123.011828

**Published:** 2023-05-31

**Authors:** Esseim Sharma, Allyson Varley, Jose Osorio, Christopher Thorne, Paul Varosy, Mark Metzl, Anil Rajendra, Saumil Oza, Gustavo Morales, Anthony Magnano, Benjamin D’Souza, Matthew Sackett, Matthew Sellers, Jose Silva, Joshua Silverstein, Jeffrey Ho, Michael Hoskins, Richard Kuk, Jorge Romero, Paul C. Zei

**Affiliations:** Cardiovascular Division, Brigham and Women’s Hospital, Boston, MA (E.S., J.R., P.C.Z.).; Heart Rhythm Clinical and Research Solutions, LLC, Birmingham, AL (A.V., J.O., C.T.).; Birmingham VA Health System, AL (A.V.).; Arrhythmia Institute at Grandview Medical Center, Birmingham, AL (J.O., A.R., G.M.).; Division of Cardiology, University of Colorado Anschutz Medical Campus, Denver (P.V.).; NorthShore University Health System, Evanston, IL (M.M.).; Department of Cardiology, Ascension St. Vincent’s Health System, Jacksonville, FL (S.O., A.M.).; Department of Medicine, Penn Presbyterian Medical Center, University of Pennsylvania, Philadelphia (B.D.).; Centra Heart and Vascular Institute, Lynchburg, VA (M. Sackett, J. Silva, R.K.).; Bon Secours St. Francis, Greenville, SC (M. Sellers).; Allegheny Health Network, Pittsburgh, PA (J. Silverstein).; Pulse Heart Institute, Tacoma, WA (J.H.).; New Mexico Heart Institute, Albuquerque (M.H.).

**Keywords:** atrial fibrillation, catheter ablation, ethics committees, research, follow-up studies, humans

While pulmonary vein isolation (PVI) has been shown to be safe and efficacious in the treatment of paroxysmal atrial fibrillation, success rates remain lower in the catheter ablation of persistent atrial fibrillation (PsAF). With a lack of randomized data to guide ablation beyond PVI in PsAF, the optimal catheter ablation strategy remains unclear. Real-world data regarding ablation strategies have not been well characterized.

The Real-AF (Real-World Experience of Catheter Ablation for the Treatment of Symptomatic Paroxysmal and PsAF Using Novel Contact Force Technologies) registry is a prospective, observational, multicenter registry of symptomatic patients undergoing radiofrequency ablation for atrial fibrillation. The Real-AF registry is funded through an investigator-initiated research grant from Biosense Webster and approved by the Western Institutional Review Board; this approval does not permit sharing of data. Data are collected over a follow-up period of 12 months. Details on the registry design and objectives have been described previously.^1^ Briefly, sites are required to have at least 1 operator with high case volume (≥100 cases per operator per year) and low fluoroscopy use (<5 minutes). Quality is maintained by source verification of 20% of the data by monitors and data collection by trained staff at each site. All patients with PsAF undergoing first-time ablation in the Real-AF registry were included in this analysis. Arrhythmia recurrences were monitored through standard-of-care practices, including event monitors as needed, EKGs at every visit, and interrogation of implanted devices if applicable. Data were collected from February 2021 to May 2022.

Patients with PsAF were separated into PVI-only and PVI+ groups for comparison. Patients in the PVI group underwent PVI alone. The PVI+ group included patients who received PVI and any other ablation. Cavotricuspid isthmus and carinal lines did not affect assignment. To describe the differences in continuous variables, means were compared using independent-sample *t* tests. A χ^2^ test was performed to describe the differences in proportions. The Benjamini-Hochberg correction was used to adjust for multiple comparisons.

The sample included 1036 index radiofrequency ablations in patients with PsAF from 51 operators at 25 participating hospitals throughout the United States. PVI+ was performed in most patients (61.5%; Table 1). Among patients undergoing PVI+, there were significantly more female patients (27.8% versus 40.7%; *P*<0.001). Patients were also significantly older (66.0±9.8 versus 70.2±8.7 years; *P*<0.001) and had a higher CHADS2Vasc score (2.9±1.6 versus 3.4±1.6; *P*<0.001). Baseline left atrial volume on fast anatomical mapping was significantly higher in PVI+ patients (108.7±32.8 versus 125.6±38.5 cm^3^; *P*<0.001). Patients undergoing PVI+ had significantly higher percentages of left atrial scar (3.1±9.0% versus 16.1±20.5%; *P*<0.001). Patients in the PVI+ group were also much more likely to present in atrial fibrillation than those in the PVI group (45.9% versus 72.5%; *P*<0.001). Total procedure time (90.7±39.4 versus 104.4±51.5 minutes; *P*<0.001) and maximum esophageal temperature (37.6±1.7 versus 38.2±1.6 °C; *P*<0.001) were greater in PVI+ ablation.

**Table 1.**
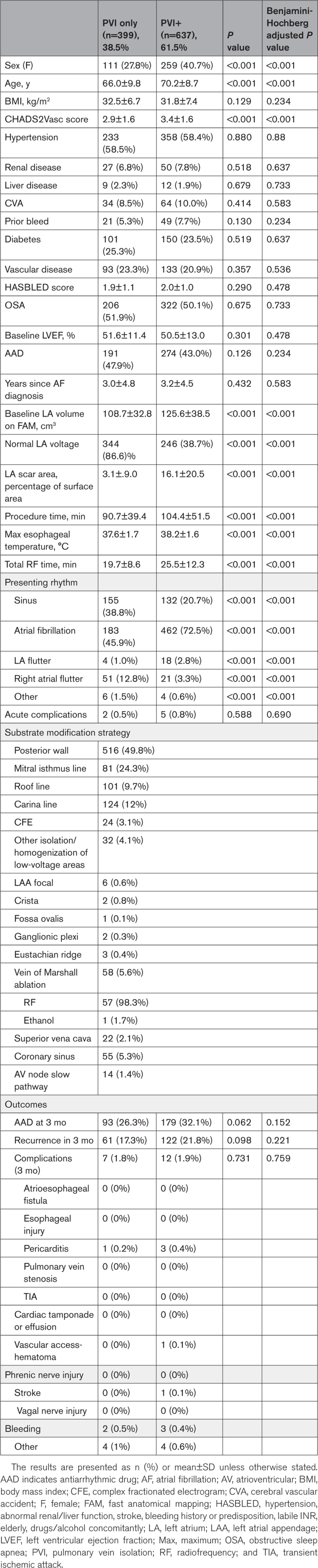
Demographics, Procedural Characteristics, and Outcomes of Patients Undergoing PVI Only vs PVI+

The most common substrate modification was left atrial posterior wall isolation, which was performed in 49.8% of patients (Table 1). Mitral isthmus (24.3%) and roof lines (9.7%) were the next most common. Vein of Marshall ablation was performed in 5.6% of patients, most performed with radiofrequency (98.2%) rather than ethanol (1.7%). Ablation targeting right-sided structures such as the superior vena cava (2.1%), slow pathway region (1.4%), or fossa ovalis (0.1%) were infrequently performed.

Finally, there were no significant differences in acute (0.5% versus 0.8%; *P*=0.690) or 3-month complications (1.8% versus 1.9%; *P*=0.759). Atrial arrhythmia recurrence and antiarrhythmic drug use were not significantly different at 3 months (17.3% versus 21.8%; *P*=0.221 and 26.3% versus 32.1%; *P*=0.152).

Given the lack of evidence to guide the best approach for PsAF ablation, our data highlight that PVI+ ablation is performed in most de novo PsAF ablations. Decisions regarding PVI+ may have been driven by left atrial substrate abnormalities on electroanatomical mapping. However, data on why specific lesion sets were chosen were not collected in the registry. Additionally, data on the location of abnormal voltage, voltage cutoffs, and mapping rhythm were not collected. While PVI+ ablation is associated with increased procedure time and esophageal temperature, it is not associated with increased acute or 3-month complications. Finally, despite the higher rate of comorbidities and substrate abnormalities in the PVI+ group, 3-month outcomes were similar.

These prospective observational registry findings are only hypothesis generating. Long-term outcome data regarding the atrial arrhythmia burden post-ablation are actively being collected and will be reported in future publications. Finally, this registry only enrolls high-volume centers with experienced operators that use limited or zero fluoroscopy as their standard approach to all ablations; thus, the results may not be applicable to all providers and centers.

## Article Information

### Sources of Funding

The Real-AF (Real-World Experience of Catheter Ablation for the Treatment of Symptomatic Paroxysmal and PsAF Using Novel Contact Force Technologies) registry is funded through an investigator-initiated research grant (primary investigator: Dr Osorio) from Biosense Webster.

### Disclosures

Dr Osorio serves as a consultant for and has received honoraria and research grants from Biosense Webster and Boston Scientific. Dr Metzl reports honoraria and consulting from Abbott, Biosense Webster, and Medtronic. Dr Rajendra reports honoraria and consulting from Biosense Webster, Philips, and Acutus Medical. Dr Oza reports honoraria and consulting from Biosense Webster. Dr Morales reports research support, consulting, and honoraria from Biosense Webster. Dr Magnano belongs to the Medtronic Advisory Board. Dr D’Souza reports honoraria and consulting from Biosense Webster, Abbott, and Stereotaxis. Dr Silverstein reports honoraria and consulting from Biosense Webster and Medtronic. Dr Romero has consulting engagements with Biosense Webster, Sanofi-Aventis, and AtriCure. Dr Zei reports research support and consulting from Biosense Webster; consulting and honoraria from Abbot; research support and consulting from Varian and Boehringer Ingelheim (expert witness); and consulting from Affera. The other authors report no conflicts.
